# Entangled: Rapunzel syndrome with postoperative intussusception

**DOI:** 10.1093/jscr/rjae623

**Published:** 2024-10-03

**Authors:** Zoha Shahzad, Osama Ijaz, Umar Akram, Neha Pervez, Asad Gul Rao, Abdulqadir J Nashwan

**Affiliations:** Department of Medicine, Fatima Jinnah Medical University, Queen's Road, Mozang Chungi, Lahore, Punjab 54000, Pakistan; Department of Medicine, Services Institute of Medical Sciences, Jail Rd, Shadman 1 Shadman, Lahore, Punjab 54000, Pakistan; Department of Medicine, Allama Iqbal Medical College, Lahore, Punjab 54700, Pakistan; Department of Surgery, Dow University of Health Sciences, Mission Rd, New Labour Colony Nanakwara, Karachi, Karachi City, Sindh 72400, Pakistan; Department of Surgery, Dow University of Health Sciences, Mission Rd, New Labour Colony Nanakwara, Karachi, Karachi City, Sindh 72400, Pakistan; Nursing and Midwifery Research Department (NMRD), Hamad Medical Corporation, PO Box 3050, Doha, Baladiyat ad Dawhah, Qatar

**Keywords:** rapunzel syndrome, trichotillomania, trichobezoar, intussusception

## Abstract

We present a case of a 7-year-old girl with Rapunzel syndrome, a rare complication of trichobezoar, initially presenting with nonspecific symptoms. Diagnostic challenges led to delayed intervention. Surgical removal of the trichobezoar was successful, but postoperative complications included jejunojejunal intussusception, highlighting the necessity for vigilant follow-up and comprehensive psychiatric evaluation. The emergence of intussusception emphasizes the profound impact of underlying psychiatric disorders, such as trichotillomania and trichophagia, which contribute to trichobezoar formation. These conditions necessitate ongoing psychiatric management to address behavioral factors that predispose to recurrent bezoar formation and associated gastrointestinal complications. Effective follow-up strategies should encompass immediate postoperative care and long-term psychiatric support to optimize patient outcomes and minimize the risk of recurrent intussusception.

## Introduction

A bezoar is a solid mass of indigestible material that accumulates in the digestive tract. Trichobezoar, specifically, is a mass composed of hair that forms in the stomach and is typically associated with trichophagia, the ingestion of hair. Other types of bezoars include phytobezoar (composed of indigestible food particles), lactobezoar (composed of milk proteins), and pharmacobezoar (composed of medication concentrates).

Rapunzel syndrome is a rare and severe complication of trichobezoar where the hair mass extends beyond the stomach into the small intestine, often reaching the jejunum or even the ileocecal junction. This syndrome was first described by Vaughan *et al.* in 1968 and remains an uncommon diagnosis in children, with fewer than 40 cases reported in the literature [1]. Rapunzel syndrome can present with a range of symptoms, from mild gastrointestinal discomfort to severe complications such as bowel obstruction and intussusception.

Most cases of trichobezoar are reported in females, likely due to the traditionally long hair in females [2]. Trichobezoars can take several years to form. After their formation, symptoms may not present immediately and are often subtle at the onset. As they grow, they may lead to more severe issues like epigastric pain, gastric outlet obstruction, ulceration, gastrointestinal bleeding, and potentially perforation [3]. Trichobezoar is caused by psychiatric disorders such as trichotillomania (the compulsion to pull out one’s hair) and trichophagia (the compulsion to eat one’s hair). These conditions predominantly affect young girls, with or without known psychiatric disorders [4].

We present the case of a 7-year-old girl with Rapunzel syndrome who presented with postprandial abdominal pain and distention, highlighting the clinical and diagnostic challenges associated with this rare condition.

## Case presentation

A 7-year-old female presented with a 3-week history of abdominal pain, distension, fatigue, and intermittent vomiting. She had reported mild epigastric discomfort present for ~8 months, along with nausea and recurrent vomiting over the past month. Physical examination revealed a palpable, firm mass in the epigastrium with minimal tenderness. The patient’s caregivers noted a history of selective eating behaviors and food aversions, prompting consideration of autism spectrum disorder or related behavioral conditions. Notably, her habit of ingesting hair had gone unnoticed. One year earlier, she had experienced pronounced hair loss and received treatment for alopecia areata with corticosteroids. Upon admission, the patient did not display signs of depression, anxiety, or intellectual disability.

While no definitive psychiatric diagnoses were made initially, the patient’s selective eating patterns and alopecia history suggest underlying psychiatric implications warranting further investigation.

## Investigations

**Figure 1:**
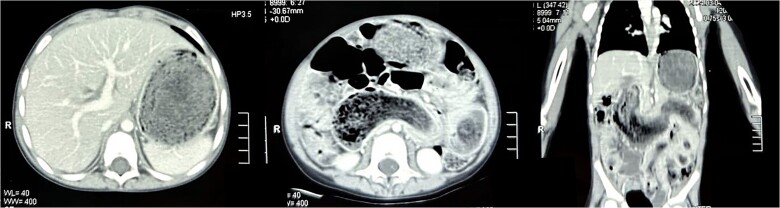
Transverse section of the CT scan of the abdomen revealing non-contrast enhancing, well-circumscribed homogenous mass in the stomach (left). Coronal section of the CT scan of the abdomen revealing the mass extending from the stomach all the way into jejunum (right).

Laboratory investigations revealed iron deficiency anemia as the only significant finding. Abdominal ultrasound demonstrated multiple gas-filled bowel loops suggestive of incomplete obstruction. Subsequently, a contrast-enhanced computerized tomography (CT) scan of the abdomen revealed gastric and small bowel distension, along with a well-circumscribed homogeneous mass exhibiting a mottled gas appearance, suggestive of the presence of air and undigested food material (Fig. 1). The intraluminal mass displayed a characteristic hairball-like pattern, extending from the stomach to the distal jejunum, with small areas of hypodensity indicative of gastric and small bowel trichobezoar, consistent with Rapunzel syndrome. Additionally, a well-defined round lesion with a swirl pattern, suggestive of intussusception, was observed near the left iliac fossa.

## Treatment

**Figure 2:**
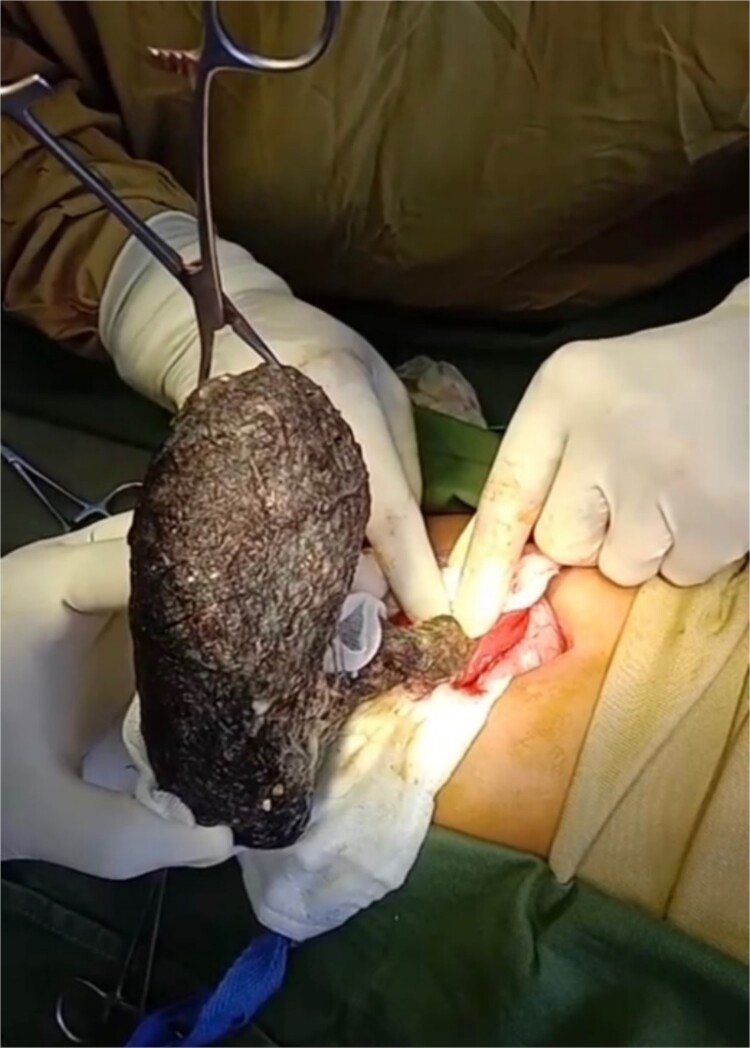
Trichobezoar being removed from the jejunum.

**Figure 3:**
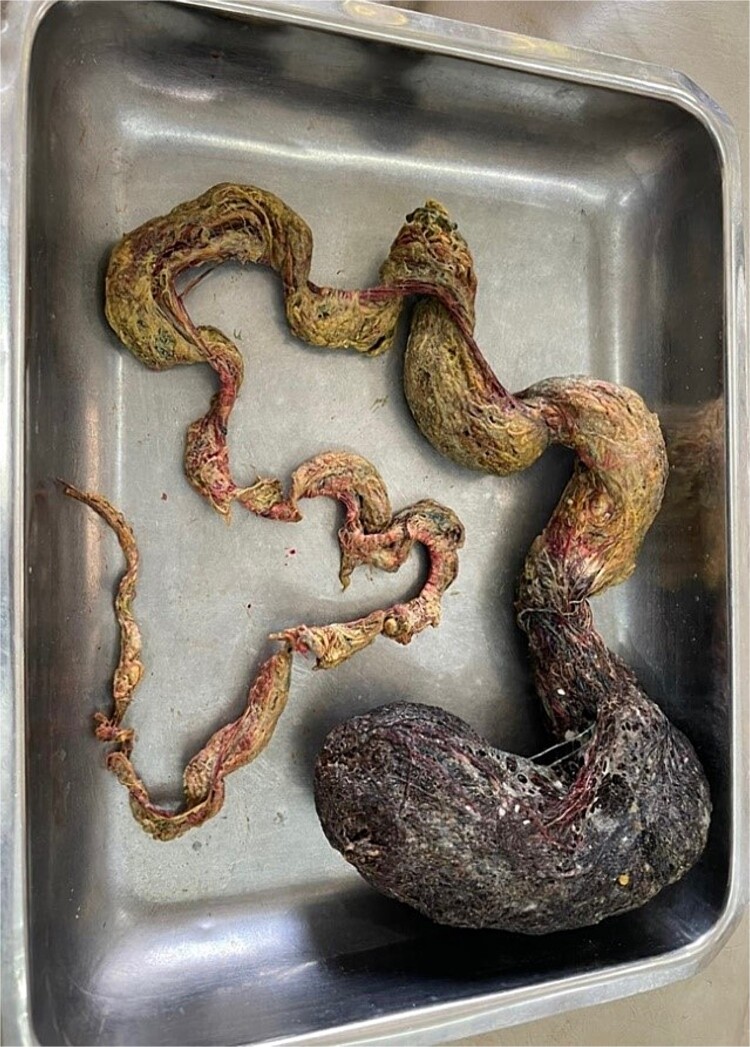
Gross specimen of the trichobezoar.

The patient underwent an elective upper midline laparotomy, during which surgeons identified a large trichobezoar measuring ~1.143 m, composed of clumped hair and cotton fibers (Fig. 2). Concurrently, ileoileal intussusception was also identified. The surgical team successfully removed the trichobezoar (Fig. 3) and reduced the intussusception. Examination of the gastric mucosa revealed minimal inflammation, prompting the primary closure of the surgical site.

## Outcome and follow-up

**Figure 4:**
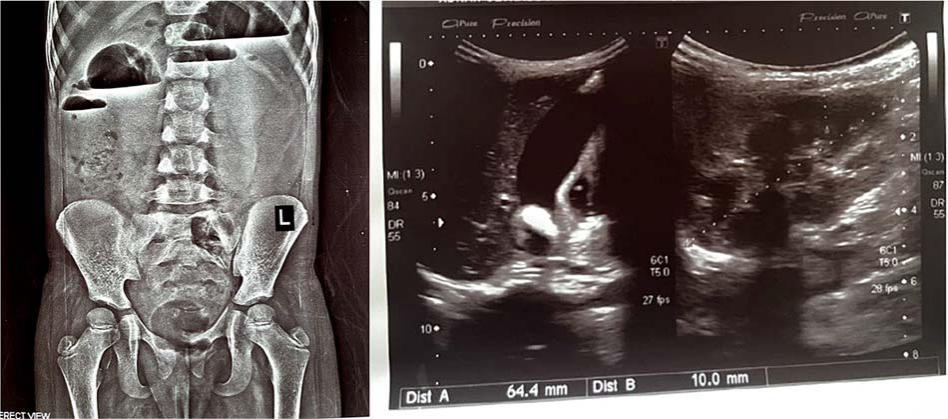
X-ray abdomen erect showing air fluid levels (left). Ultrasound abdomen showing intussusception (right).

The patient experienced an uneventful postoperative course during her hospital stay and was discharged home with scheduled outpatient psychiatric follow-up. However, she presented again after 3 weeks with severe abdominal pain, tenderness, and recurrent vomiting. Imaging studies, including abdominal X-rays and ultrasounds, revealed the presence of air-fluid levels (Fig. 4, left) and confirmed intussusception (Fig. 4, right)*.* Attempts to resolve the intussusception through non-surgical means, such as enema reduction, were unsuccessful. Consequently, a repeat upper midline laparotomy was performed, revealing jejunojejunal intussusception, which was successfully managed through manual reduction. The recurrent intussusception was determined to be attributable to postoperative adhesions.

## Discussion

Trichobezoars, characterized by the accumulation of hair in the stomach, often conform to the gastric cavity’s shape. Trichophagia, the compulsive ingestion of hair, initiates their formation by trapping hair in gastric mucosal folds, leading to gradual enlargement [2]. The prevalence of trichotillomania, a psychiatric disorder characterized by hair-pulling behavior, ranges from 0.6 to 1.6% according to DSM-IV criteria. Notably, ~30% of individuals with trichotillomania also exhibit trichophagia, with ~1% of these cases requiring surgical intervention [5]. The co-occurrence of trichotillomania and trichophagia may also manifest in alopecia, underscoring their relevance in pediatric alopecia evaluations.

Rapunzel syndrome, an uncommon yet severe complication of trichobezoars, refers to the extension of a bezoar beyond the pyloric sphincter, forming a tail-like structure that can extend into the jejunum or further to the ileocecal junction [6]. Initially asymptomatic, Rapunzel syndrome can progress with symptoms such as abdominal pain, nausea, vomiting, bowel obstruction, weight loss, anorexia, hematemesis, and potentially life-threatening conditions like peritonitis or intussusception [2]. Complications associated with large or obstructing bezoars include gastric ulceration, obstructive jaundice, acute pancreatitis, and malabsorption-related conditions such as protein-losing enteropathy, iron deficiency, and megaloblastic anemia [7, 8].

In the current case, the patient remained asymptomatic for an extended period, with undetected underlying trichotillomania and trichophagia leading to delayed intervention until the development of Rapunzel syndrome. Initially attributed to other causes such as alopecia, the patient’s hair loss was not immediately linked to behavioral abnormalities due to the absence of overt signs observed by caregivers.

Diagnosing gastric bezoars presents challenges, as they may not always be visualized on abdominal ultrasound due to the high echogenicity of the hair and trapped air bubbles. Contrast-enhanced CT scans, offering diagnostic accuracy ranging from 73 to 95%, are preferred for detecting trichobezoars [9]. However, upper gastrointestinal endoscopy remains the gold standard for diagnosis, providing direct visualization and therapeutic capabilities, especially for smaller bezoars [2].

Surgical intervention becomes necessary when large or solid bezoars cause perforation, hemorrhage, or extensive extension into the intestinal tract, as seen in Rapunzel syndrome. While traditional approaches involved upper midline laparotomy for bezoar extraction, modern techniques like laparoscopy are increasingly favored, particularly for smaller to moderate-sized bezoars [2]. Additional methods such as extracorporeal shock wave lithotripsy, intragastric enzyme administration, and pharmacological interventions have demonstrated varying success in managing bezoars [2, 10].

Postoperative psychiatric follow-up and parental counseling are crucial for preventing recurrence, even without overt behavioral abnormalities. Recommending postoperative surgical follow-up and advising parents to seek evaluation for any postoperative abdominal pain are imperative.
